# Biocatalytic reduction of alkenes in micro-aqueous organic solvent catalysed by an immobilised ene reductase†

**DOI:** 10.1039/d3cy00541k

**Published:** 2023-05-22

**Authors:** Rocio Villa, Claudia Ferrer-Carbonell, Caroline E. Paul

**Affiliations:** a Biocatalysis section, Department of Biotechnology, Delft University of Biotechnology van der Maasweg 9 2629 HZ Delft The Netherlands c.e.paul@tudelft.nl

## Abstract

Biocatalytic asymmetric reduction of alkenes in organic solvent is attractive for enantiopurity and product isolation, yet remains under developed. Herein we demonstrate the robustness of an ene reductase immobilised on Celite for the reduction of activated alkenes in micro-aqueous organic solvent. Full conversion was obtained in methyl *t*-butyl ether, avoiding hydrolysis and racemisation of products. The immobilised ene reductase showed reusability and a scale-up demonstrated its applicability.

## Abstract 2

Immobilised *Ts*OYE on Celite carrier catalyses the bioreduction of activated alkenes in pure organic solvent, providing full conversion to (chiral) products.

## Graphical abstract

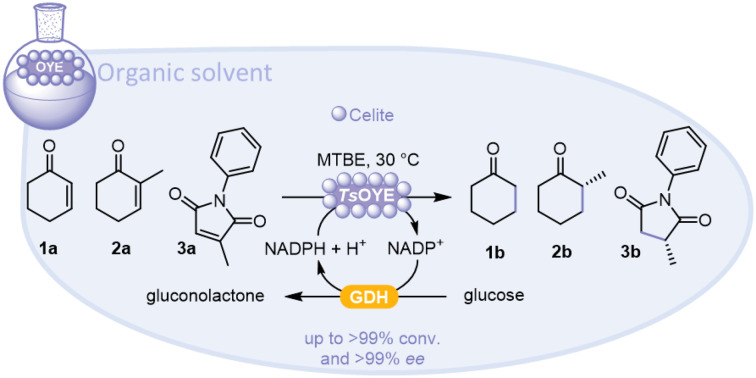

Chiral substituted alkanes form the framework of many natural products and are key chemical building blocks, mainly produced by asymmetric reduction catalysed by transition metals.^1^ However, high selectivity is not always achieved, and can alternatively be attained using biocatalysts.^2^ In this respect, ene reductases from the Old Yellow Enzyme family (OYEs) are ideal for asymmetric reductions of alkenes, displaying exquisite selectivity under mild reaction conditions.^3–5^ OYEs are nicotinamide adenine-dinucleotide NAD(P)H-dependent and contain a prosthetic flavin mononucleotide (FMN) to reduce activated alkenes (*e.g.* α,β-unsaturated aldehyde, ketone, acid, ester, nitro and nitrile)^4^ to the corresponding alkane product with up to two stereogenic centres (Fig. 1A). The performance of these biocatalysts is promising because of their high regio-, stereo-, and chemoselectivity, an expanding enzyme portfolio, substrate scope and type of chemical reactions they can catalyse.^6–8^ Currently, more than 60 OYEs are characterised from plantae, fungi, bacteria, algae and cyanobacteria, divided across different classes.^4,7^ Class III represents thermophilic-like OYEs, among which the thermostable *Ts*OYE (formerly CrS), isolated from *Thermus scotoductus* SA-01,^9^ catalyses a wide range of activated alkene substrates and has potential for scale-up.^10^

**Fig. 1:**
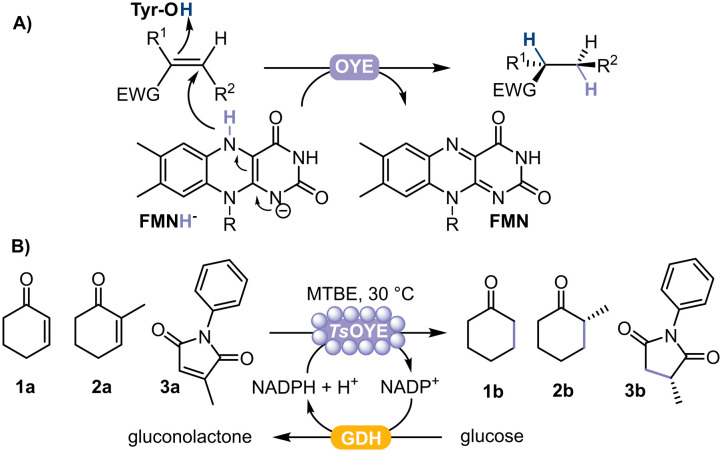
A) Schematic representation of the asymmetric reduction of activated alkenes catalysed by flavin-dependent OYE. EWG = electron withdrawing group. B) Reduction of cyclohexenone 1a, 2-methylcyclohexenone 2a, or 2-methyl-*N*-phenylmaleimide 3a catalysed by immobilised *Ts*OYE on Celite with GDH as cofactor recycling system.

Yet the use of OYEs in industrial processes has been generally restricted, in part due to low product titers as a consequence of the poor solubility of alkene substrates in water and racemisation of chiral products.^11–13^ To address these issues, water immiscible organic solvents can be used to form biphasic or even micro-aqueous systems. OYE reactions in these micro-aqueous media would avoid the drawbacks in water, such as low substrate solubility, hydrolysis, product racemisation and mass transfer limitations.^14^ Oxidoreductases in general require a minimum water content (*a*_w_ 0.1–0.7) to achieve 10% of their activity.^15^ The reduction of cyclohexenone catalysed by free OYE1 (from *Saccharomyces pastorianus*) in micro-aqueous organic media has been reported,^16^ with different solvents of varying partition coefficient log *P* values. A log *P* value ≥1.8 at ≥90% v/v achieved full conversion. Biphasic systems were also shown to improve the stereoselectivity of OYE-catalysed reactions,^17,18^ and facilitate their separation and recycling from water insoluble products.^19^

In parallel, immobilisation of OYEs can improve mass transfer limitations and product isolation, and allow straightforward separation from the reaction media and further reuse.^20^ Previous immobilisation of OYE3 (from *Saccharomyces cerevisiae*) by covalent tethering with glyoxyl-agarose (GA) or by affinity-based adsorption on EziG™ has been demonstrated in buffer at 5 mM substrate concentration with low recyclibility.^21^ The most promising example includes OYE3 and NCR (from *Zymomonas mobilis*) each co-immobilised with a glucose dehydrogenase (GDH) from *Bacillus megaterium* on a glutaraldehyde-activated Relizyme HA403/M carrier.^22^ Moderate conversions with 88–97% ee values were achieved on preparative scale with co-solvents. Unfortunately absorption of product led to poor product recovery, and washing with a solvent deactivated the biocatalyst for subsequent cycles. Another example includes co-immobilisation of YqjM (from *Bacillus subtilis*) with a GDH as cross-linked enzyme aggregates (CLEAs),^23^ however reactions were also performed in buffer, requiring product extraction.

In this study, we selected *Ts*OYE for immobilisation by adsorption on different Celite supports to catalyse the asymmetric reduction of cyclohexenone 1a, 2-methylcyclohexenone 2a and 2-methyl-*N*-phenylmaleimide 3a in a micro-aqueous organic solvent system (Fig. 1B).

We started by exploring the conversion of cyclohexenone 1a with free *Ts*OYE in four water immiscible organic solvents, toluene, ethyl acetate (EtOAc), methyl *t*-butyl ether (MTBE) and heptane (Fig. 2). The reactions contained 8.3% v/v water due to the addition of the required NADPH cofactor, glucose, a GDH double mutant E170K/Q252L from *Bacillus subtilis* (*Bs*GDH)^24^ and *Ts*OYE in buffer solution. As a control, buffer-saturated MTBE (*a*_w_ 1.0) was also used. High conversions of 85 ≥ 99.9% were obtained with all four solvents (Fig. 2).

**Fig. 2:**
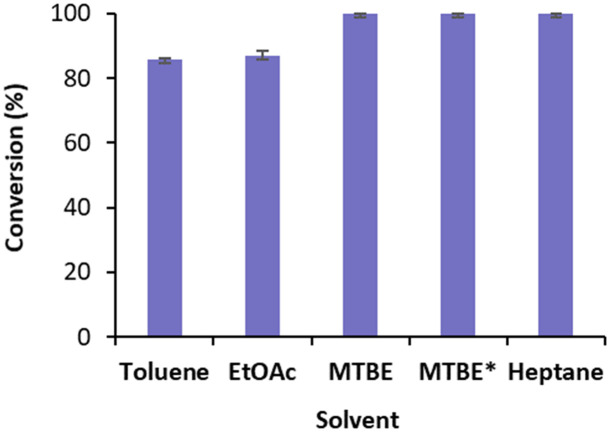
Conversions for the reduction of cyclohexenone 1a catalysed by free *Ts*OYE in organic solvents. Conditions: 10 mmol 1a, 10 U *Bs*GDH, 20 mmol glucose, 0.2 mmol NADPH, 1.4 μmol *Ts*OYE, organic solvent (91.7% v/v, 8.3% v/v buffer), 900 rpm, 30 °C, 24 h. *Buffer-saturated MTBE. Data are average of duplicates (see ESI† Table S2).

These initial results are comparable to the tolerance of organic solvents displayed by XenA (*Pseudomonas putida*),^17^ also belonging to class III thermophilic OYEs, and to that of OYE1 and YersER (from *Kluyveromyces lactis*).^25^ The solvent tolerance of *Bs*GDH E170K/Q252L was previously reported.^26^ The highest conversion with MTBE and heptane led us to further investigate MTBE with immobilised *Ts*OYE. In terms of carrier, calcined diatomaceous earth trademarked as Celite is considered an environmental friendly material for enzyme immobilisation with medium permeability ideal for biocatalytic reactions in continuous flow process.^27^ We selected these porous Celite as carriers for their high water adsorption capacity, which enables the control of local aqueous environment around the enzyme in organic solvents, as was shown with lyases.^28^ In addition, enzyme immobilisation by adsorption avoids the use of chemicals and covalent linkers, which can affect enzymatic activity and stability.^29^ Immobilisation of *Ts*OYE was performed by adsorption on Celite 545, R-632, R-633 and R-648 (see ESI† Table S1). As revealed by protein concentration measurements of the supernatant, all Celite showed immobilisation yields ranging from 54 to 59% (Table 1).

**Table 1:** Obtained values for the *Ts*OYE immobilisation on different Celite^a^

Celite	*Ts*OYE_0_^b^ (mg)	Immob. *Ts*OYE (mg)	*Ts*OYE/Celite (mg g^−1^)	Yield (%)
545	2.5 ± 0.1	1.4 ± 0.1	6.8 ± 0.5	54.0 ± 5.6
R-632	2.6 ± 0.1	1.4 ± 0.1	6.7 ± 0.3	53.0 ± 6.3
R-633	2.5 ± 0.1	1.5 ± 0.1	7.4 ± 0.5	58.2 ± 6.3
R-648	2.6 ± 0.1	1.5 ± 0.1	7.6 ± 0.6	58.6 ± 0.6

a Conditions: 5 h at 20 °C.

b *Ts*OYE_0_: mg of enzyme before immobilisation.

Next, the immobilised *Ts*OYE-Celite were evaluated in different reactions conditions. The first model reaction was the reduction of cyclohexenone 1a catalysed by *Ts*OYE on Celite 545 in buffer at pH 7.0, obtaining full conversion after 24 h. However, enzymatic activity of the supernatant at the end of the reaction confirmed the desorption of the enzyme in buffer. Control reactions with Celite in the absence of enzyme led to no product formation (ESI† Table S3).

Reactions with *Ts*OYE on Celite 545 were carried out in EtOAc and MTBE with 7.3% v/v buffer due to addition of NADP^+^ cofactor, glucose, and GDH solutions in buffer, affording >99.9 conversion (see ESI†). However, desorption of the enzyme was observed after the reaction. Therefore, water content was decreased through the addition of solid glucose, the lyophilised industrial grade GDH-101 (from Johnson Matthey), a concentrated cofactor stock solution and hydrated salt pairs (*i.e.* Na_2_HPO_4_·*x*H_2_O), comparing different Celite carriers (Table 2, entry 1–20).^30^

**Table 2:** Reaction conditions for the reduction of cyclohexenone 1a, 2-methylcyclohexenone 2a and 2-methyl-*N*-phenylmaleimide 3a catalysed by immobilised *Ts*OYE on Celite in MTBE^a^

Entry	[Substrate] (mmol)		[NADP^+^] (mmol)	[Glucose] (mmol)	Hydrated salt (mg)	Buffer (% v/v)	Celite carrier	*Ts*OYE-Celite (mg)	Conv. (%)	ee (% *R*)
1	1a	10	0.4	27.7	25	0.4	545	15	39.0 ± 4.2	—
2	1a	10	1	27.7	25	1	545	15	88.0 ± 9.2	—
3	1a	10	1	27.7	25	1	R-632	15	95.1 ± 0.1	—
4	1a	10	1	27.7	25	1	R-633	15	>99.9	—
5	1a	10	1	27.7	25	1	R-648	15	>99.9	—
6^b^	1a	10	1	27.7	25	1	R-633	15	94.4 ± 1.4	—
7	1a	50	1	60	25	1	R-633	40	8.0 ± 2.4	—
8	1a	50	1	60	25	4	R-633	40	>99.9	—
9	1a	50	1	60	10	4	R-633	40	96.2 ± 1.8	—
10	1a	50	1	60	25	4	R-648	40	>99.9	—
11	2a	10	1	27.7	25	2	545	40	72.1 ± 3.6	97.1 ± 0.1
12^c^	2a	10	1	27.7	25	2	545	40	59.7 ± 8.6	97.9 ± 2.9
13	2a	10	1	27.7	—	10	545	40	96.6 ± 2.2	95.5 ± 0.6
14^c^	2a	10	1	27.7	—	10	545	40	98.0 ± 1.4	94.6 ± 0.6
15	3a	10	1	27.7	25	1	R-633	40	35.5 ± 9.2	>99.9
16	3a	10	1	27.7	25	4	R-633	40	71.7 ± 6.6	>99.9
17^d^	3a	10	1	27.7	25	4	R-633	40	>99.9	>99.9
18^d^	3a	50	2	60	25	4	R-633	60	51.5 ± 2.1	>99.9
19^d^	3a	50	2	60	—	10	R-633	60	>99.9	>99.9
20^d^	3a	50	2	60	—	10	R-648	60	>99.9	>99.9

a Conditions: *Ts*OYE immobilised on Celite, 2 mg GDH-101 (20.5 U mg^−1^), anhydrous glucose, NADP^+^, hydrated salt Na_2_HPO_4_·12H_2_O/Na_2_HPO_3_·5H_2_O (1 : 1 w/w), buffer (50 mM MOPS-NaOH pH 7.0), MTBE, substrate added with 1% v/v DMSO, 1 mL volume, 24 h at 30 °C, 900 rpm in an Eppendorf Thermomixer C.

b Buffer-saturated MTBE (50 mM MOPS-NaOH pH 7.0).

c No DMSO.

d Shaking at 180 rpm in a New Brunswick Scientific Excella E24 Incubator Shaker Series. All samples in duplicates analysed by (chiral) GC-FID.

In general, *Ts*OYE on Celite 545, R-632, R-633, or R-648, gave moderate to high conversions for the reduction of cyclohexenone 1a (Table 2, entry 1–6), 2-methylcyclohexenone 2a (entry 11–14), and 2-methyl-*N*-phenylmaleimide 3a (entry 15–20) in MTBE (Fig. 1B). With *Ts*OYE-Celite 545 with a buffer amount as low as 0.4% v/v in MTBE and 0.4 mmol cofactor, the conversion was lower (39%, Table 2 entry 1), than with 1% v/v buffer with 1 mmol cofactor (88%, entry 2). Buffer-saturated MTBE with *Ts*OYE-Celite R-633 showed maximum water activity was reached with 1% v/v water (entry 4 *vs.* 6).

Fig. 3 depicts the time-course conversion for the reduction of cyclohexenone 1a catalysed by *Ts*OYE-Celite 545, R-632, R-633 and R-648 in MTBE with 1% v/v buffer, with close to full conversion obtained after 6 h. The slight variation in conversion rates is ascribed to the slight difference in *Ts*OYE adsorbed on the Celite carrier and the Celite capacity for water adsorption (Table 1 and ESI† Table S1).

**Fig. 3:**
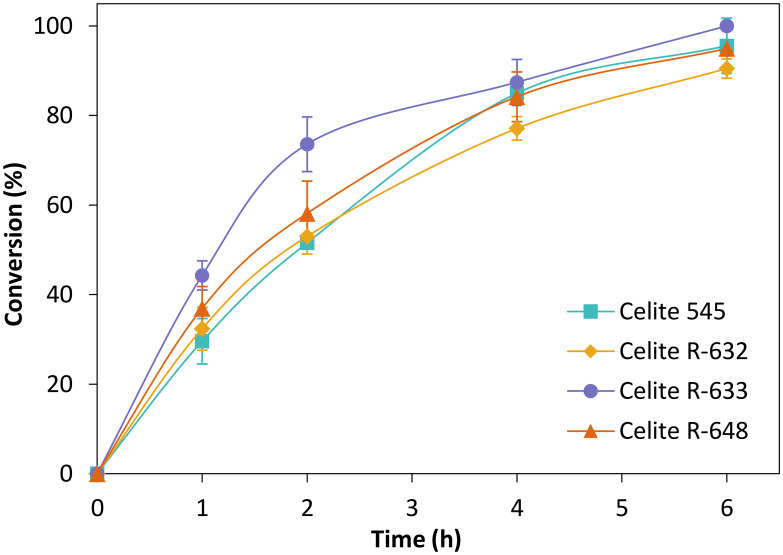
Reduction of cyclohexenone 1a catalysed by immobilised *Ts*OYE in MTBE. Conditions: 50 mg *Ts*OYE-Celite, 10 mmol 1a, 1 mmol NADP^+^, 27.7 mmol glucose, 25 mg Na_2_HPO_4_·12H_2_O/Na_2_HPO_3_·5H_2_O, 2 mg GDH-101, MTBE, 1 mL volume, 900 rpm, 30 °C.

Increasing substrate concentration to 50 mmol cyclohexenone, with 40 mg of *Ts*OYE-Celite R-633 and R-648, required increasing the buffer content from 1 to 4% v/v to reach conversions of 96 ≥ 99% (Table 2, entry 7 *vs.* 8–10). We ascribe this water requirement to several factors: the solubility of glucose, as higher substrate concentrations increase the amount needed for cofactor recycling; the solubility of the cofactor; the reaction mechanism itself for protonation of the product (Fig. 1A). For the latter, more extensive mechanistic investigations of the intricate proton shuttling in OYEs would be interesting, as previous studies demonstrated the importance of the active site tyrosine residue as a source of proton,^31,32^ yet protonation can still occur even when switching this tyrosine for a phenylalanine, albeit at lower rate.^9^ We also observed that adding 25 mg of hydrated salt pairs was superior to 10 mg (Table 2, entry 8 *vs.* 9) when using 4% v/v buffer content.

Regarding enantiomeric excess, the obtained ee values of (*R*)-2-methylcyclohexanone 2b were improved from 85.5% ee in buffer (Fig. S13†), to 96.4–97.9% ee in MTBE (Table 2, entry 11–14). This difference is ascribed to racemisation in buffer observed across these α-substituted carbonyl products, such as in cyclic ketones^13,33^ and arylpropanals.^34^ The minimal aqueous content in the MTBE media provided higher ee values, therefore, we also observed racemisation of the product 2b was limited with 2% v/v buffer compared with 10% (entry 11 *vs.* 13). The influence of DMSO (1% v/v) on the reaction showed an increase in conversion (entry 11 *vs.* 12), most probably due to its water content. It should be noted that we cannot exclude adsorption of the GDH-101 on the carrier during the reaction. The lower water content may also limit hydrolysis of gluconolactone to gluconic acid.

Subsequently, the reduction of 2-methyl-*N*-phenylmaleimide 3a was explored by varying buffer content in MTBE (Table 2, entry 15–20). In this case, 4% v/v buffer was needed for 10 mmol substrate and 10% v/v for 50 mmol. We also observed a strong influence of shaking, with a conversion of 71% when running the reaction in a thermomixer, compared with >99% in an incubator shaker with a wider rotation angle. This difference may be due to the homogenisation of the reaction mixture as the Celite easily pooled at the bottom of the reaction vial.

Fig. 4 depicts the time-course conversion for the reduction of 2-methyl-*N*-phenylmaleimide 3a catalysed by *Ts*OYE-Celite R-633 in MTBE in a thermomixer compared with an incubator platform. Conversion close to 80% and >99% were obtained after 6 h, respectively, demonstrating the requirement for adequate shaking for this reaction system.

**Fig. 4:**
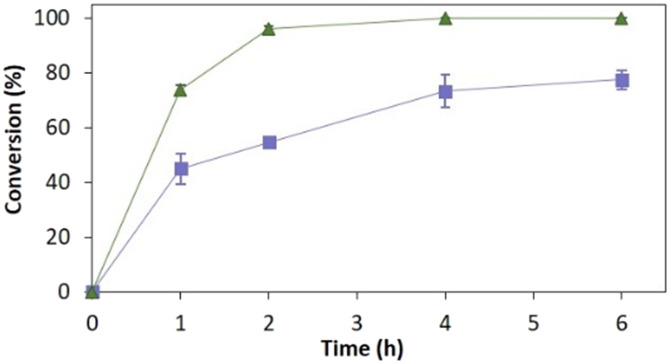
Reduction of 2-methyl-*N*-phenylmaleimide 3a catalysed by immobilised *Ts*OYE in MTBE. Conditions: 50 mg *Ts*OYE-Celite R-633, 10 mmol 3a, 1 mmol NADP^+^, 27.7 mmol glucose, 25 mg Na_2_HPO_4_·12H_2_O/Na_2_HPO_3_·5H_2_O, 2 mg GDH-101, MTBE, 1 mL volume, 30 °C. Blue squares: 900 rpm in a thermomixer; green triangles: 180 rpm in an incubator shaker.

Taking these conditions into account, scale-up reactions with 50 mmol 2-methyl-*N*-phenylmaleimide 3a gave full conversion with *Ts*OYE-Celite R-633 and R-648 with 10% v/v buffer (Table 2, entry 19–20). The product was easily isolated by separation and evaporation of the organic phase, affording 91% of (*R*)-2-methyl-*N*-phenylsuccinimide 3b, avoiding extraction difficulties and hydrolysis typically obtained in aqueous media.^35,36^ The isolated product was analysed by GC-FID, ^1^H- and ^13^C-NMR and proved to be of >98% purity (see ESI†). The turnover number of the reaction was estimated at *ca.* 4200.

The influence of hydrated salt pairs and enzyme reusability was then evaluated with *Ts*OYE-Celite 545 and R-633 (Fig. 5). Reactions with and without salt pairs both provided >99% conversion after 24 h in the first cycle. For the second and third operational cycle, reactions with salt pairs added at each cycle (Fig. 5, blue), outperformed those with salt pairs added in the first cycle only (Fig. 5, green). Reactions carried out in the absence of salt pairs (Fig. 5, yellow) gave close to complete loss of conversion after the second operational cycle. Thus, we observed a clear influence of salt pairs to control the aqueous environment of the enzyme at each operational cycle. Interestingly, Celite 545 seemed to perform better than Celite R-633 in reactions without salt pairs (yellow), and with salt pairs added in the first cycle only (green). This result can be attributed to higher retention of water for Celite 545 (Table S1†).

**Fig. 5:**
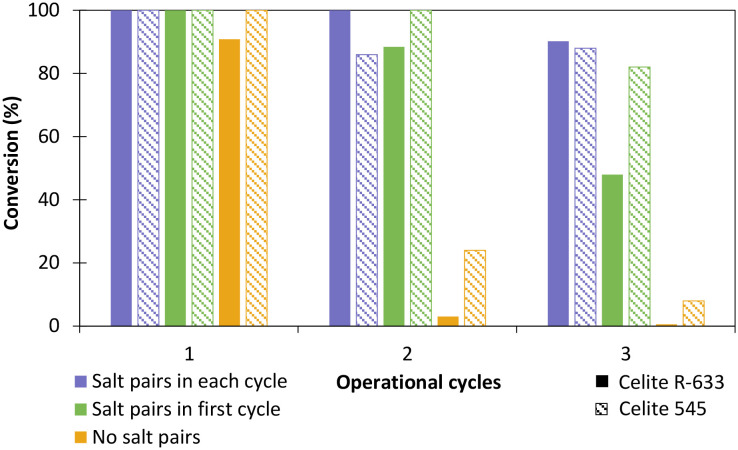
Influence of salt pairs on the reduction of cyclohexenone 1a catalysed by immobilised *Ts*OYE in MTBE. Conditions: 10 mmol 1a, 1 mmol NADP^+^, 2 mg GDH-101, 27.7 mmol glucose, 25 mg Na_2_HPO_4_·12H_2_O/Na_2_HPO_3_·5H_2_O or no salt pairs (yellow), 50 mg *Ts*OYE-Celite R-633 (full pattern) or 545 (stripped pattern), MTBE (2% v/v buffer content), 1 mL volume, 900 rpm, 30 °C, 24 h.

Once the influence of the salt pairs established, *Ts*OYE-Celite R-633 was further selected to evaluate reusability over multiple operational cycles (Fig. 6). Full conversion was obtained after the second cycle, and *ca.* 70% conversion after five consecutive cycles. The slight systematic decrease in conversion after the third cycle could be explained by potential loss of immobilised enzyme material when removing the supernatant after each reaction.

**Fig. 6:**
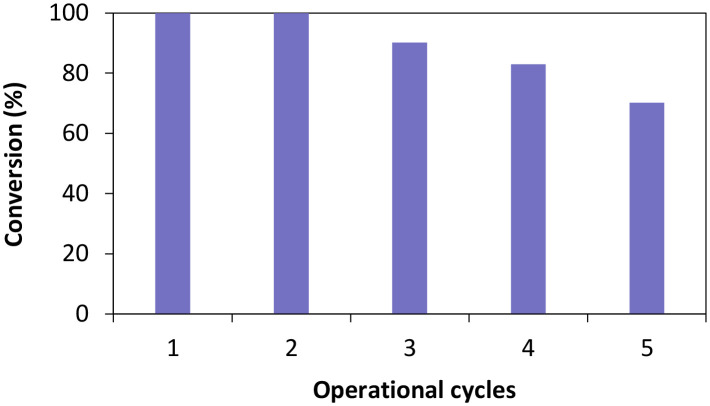
Operational cycles of *Ts*OYE immobilised on Celite R-633 for the reduction of cyclohexenone 1a in MTBE. Conditions: 50 mg *Ts*OYE-Celite R-633, 10 mmol 1a, 1 mmol NADP^+^, 2 mg GDH-101, 27.7 mmol glucose, 25 mg Na_2_HPO_4_·12H_2_O/Na_2_HPO_3_·5H_2_O, MTBE (1% v/v buffer content), 1 mL volume, 900 rpm, 30 °C, 24 h each cycle.

In summary, immobilisation of *Ts*OYE on Celite by adsorption provided an active, stable and reusable biocatalyst for the reduction of activated alkenes in micro-aqueous organic solvent. Due to the great interest in these enzymes for preparative applications, access to heterogeneous OYEs allows enzyme recycling, easier product separation, and minimises hydrolysis and racemisation that plague substituted saturated carbonyl products in aqueous media. Further developments to fully exploit this system remain to be investigated, for example for applications in continuous flow systems with cofactor recycling or enzyme coupling in a cascade fashion.

## Author contributions

R. V. and C. F.-C. performed the experiments; R. V. analysed the results and wrote the first draft of the manuscript; C. E. P. conceptualised and supervised the study, reviewed and edited the manuscript. All authors have read and agreed to the published version of the manuscript.

## Conflicts of interest

There are no conflicts to declare.

## Supplementary Material

CY-013-D3CY00541K-s001
