# Supplementary material for: Biocatalytic reduction of alkenes in micro-aqueous organic solvent catalysed by an immobilised ene reductase
Source: Catal Sci Technol. 2023 May 22;13(19):5530–5. doi: 10.1039/d3cy00541k (PMC10544049; doi:10.1039/d3cy00541k)
Supplement: CY-013-D3CY00541K-s001 [file CY-013-D3CY00541K-s001.pdf]

Electronic Supplementary Material (ESI) for Catalysis Science & Technology.
This journal is © The Royal Society of Chemistry 2023

Supporting information

Biocatalytic reduction of alkenes in micro-aqueous organic solvent catalysed by an immobilised ene reductase

Rocio Villa, Claudia Ferrer-Carbonell, and Caroline E. Paul*

Biocatalysis section, Department of Biotechnology, Delft University of Biotechnology, van der Maasweg 9, 2629 HZ Delft, The Netherlands

Contents

General information 2
*Ts*OYE production and purification 2
Enzyme concentration and activity 3
Enzyme immobilisation 3
Biotransformations 3
Free enzyme 3
Immobilised enzyme 4
Control experiments 4
Scale-up 5
Immobilised enzyme recovery 5
Product analyses 5
GC chromatograms 7
References 13

General information

Chemicals: NADPH and $NADP^+$ were purchased from Prozomix. All other chemicals were purchased from Sigma–Aldrich, abcr GmbH or TCI Europe at the highest purity available and used as received.
Enzyme: GDH-101 was obtained from Johnson Matthey (London, England), as a lyophilised powder with an activity of 20.5 U/mg.
Celite: Celite® R-632, R-633 and R-648 were received *via* Dr. Y. Guiavarc'h from Imerys (France) (Table S1). Celite™ 545 was purchased from Thermo Scientific™.

Table S1. Physical properties of Celite carriers

Celite	Form	Mean pore diameter (µm)	Surface area, m^2/g	Total pore volume, cc/g	Volume fraction, 1.0-50 µ, cc/g	Water adsorption, % by weight, pellet method
R-632	Sphere (14/30)	7.0	2.0	1.19	1.09 (92.0%)	84
R-633	Sphere (30/50)	6.5	1.3	1.47	1.42 (96.2%)	240
R-648	Sphere (30/50)	0.14	46.0	1.18	0.39 (33.2%)	160

Celite	Product type	Mean pore size (µm)	Permeability, D^2	Estimated water (%)	Wet density, DCF
545	Flux calcined	17.0	3.0	340	20.0

*Ts*OYE production and purification

*Ts*OYE (from *Thermus scotoductus* SA-01, accession number B0JDW3) was recombinantly produced in *E. coli* BL21(DE3) cells with the plasmid pET-22b(+)-*tsoye*. A 100 mL pre-culture of LB medium containing 100 µg mL^{-1} of ampicillin was inoculated with a glycerol stock of *E. coli* BL21(DE3)-pET-22b(+)-*tsoye* and incubated overnight at 37 °C and 180 rpm. 500 mL of TB medium supplemented with 100 µg mL^{-1} of ampicillin in a 2 L shake flask was inoculated with the pre-culture (5% v/v) and incubated at 37 °C and 180 rpm until the OD_{600} reached 0.6 (approximately 2 h 30 min), 0.1 mM of IPTG was added for induction, and the cell culture was incubated overnight at 30 °C and 180 rpm.

Cells were harvested by centrifugation at 17,500 × *g* for 30 min at 4 °C. The resulting cell pellet was washed and re-suspended in a 20 mM MOPS-NaOH buffer at pH 7.0 supplemented with a spatula tip of DNase I, $MgCl_2$, and one tablet of EDTA-free Complete™ protease inhibitor. The cell pellet was re-suspended in buffer and lysed using a Multi-Shot Cell Disruption System (Constant Systems Ltd, Daventry, UK) over two cycles. Cell debris were separated from the crude extract by centrifugation at 17,500 × *g* for 30 min at 4 °C. The supernatant was filtered.

Heat purification was performed by incubating the supernatant in 50 mL Greiner tubes for 1 h 30 min in a water bath at 70 °C. Precipitated proteins were removed by centrifuging at 38,500 × *g* for 30 min at 4 °C two times. A clear and bright yellow solution of *Ts*OYE was obtained, supplemented with flavin mononucleotide (FMN) and incubated on ice for 30 min. The protein solution was concentrated using an Amicon® Ultra-15 Centrifugal Filter Device (molecular cut-off 30 kDa) and washed with 20 mM MOPS-NaOH pH 7.0 buffer until the flow-through was colourless. The resulting heat-purified *Ts*OYE was flash frozen in liquid nitrogen and stored at -80 °C until later use.

Enzyme concentration and activity

Total protein concentration was measured with a Bradford assay using bovine serum albumin (BSA).[1] The purified *Ts*OYE stock solution used for immobilisation was determined to be 152 µM.

Concentration of flavin-bound *Ts*OYE was determined by UV-Vis absorbance following standard protocol for flavoproteins (using FMN extinction coefficient at 446 nm ε_{446} = 12.2 $mM^{-1}cm^{-1}$),[2] with the following compounds and concentrations: 1 mL buffer 20 mM MOPS-NaOH pH 7.0; *Ts*OYE (amount to reach an absorbance between 0.1 and 0.2), and 20 µL sodium dodecyl sulfate (SDS, 0.2% w/v final, from a stock solution of 10% w/v in MilliQ). Enzyme purity was assessed by SDS-PAGE and estimated to be >90% pure.

The specific activity of *Ts*OYE for cyclohexenone was 9 U/mg, measured following the consumption of NADPH at 340 nm (ε = 6.22 $mM^{-1}cm^{-1}$) as described previously.[3, 4] The assay mixtures contained final concentrations of 50 mM MOPS-NaOH buffer pH 7.0, 10 mM cyclohexenone, 0.2 mM NADPH, at 22.6 °C, 1 mL in volume, all components were thermostated prior to measurements.

Enzyme immobilisation

Enzyme immobilisation on celite was performed according to a previous protocol with slight modifications.[5] In this work, 200 mg of Celite carrier were washed three times with buffer 50 mM MOPS-NaOH pH 7.0. Then, 500 µL of the enzyme *Ts*OYE were added to each of the washed carrier. An aliquot of 70 µL was taken at t = 0 h for reference. The enzyme-carrier mixture was slowly shaken for 5 h at 20 °C. After 5 h, each sample was centrifuged, the supernatant was removed, and the immobilised enzyme was frozen. Enzyme concentration before and after immobilisation was determined by UV-Vis absorbance as described above.[2]

Biotransformations

Biotransformations were performed with 1 mL reaction volume in 2 mL microcentrifuge tubes.

Free enzyme

For reactions with free *Ts*OYE (Figure 2, Table S2), the reactions were set up with the corresponding organic solvent, 1.4 µM *Ts*OYE, 0.2 mmol NADPH, 10 U/mL *Bs*GDH, 20 mmol glucose, 10 mmol cyclohexenone, 1 mL in volume, at 30 °C and 900 rpm in an Eppendorf ThermoMixer C for 24 h.
As a control, buffer saturated MTBE, using 50 mM MOPS-NaOH pH 7.0, was used in the reaction conditions described above.

For reaction with free *Ts*OYE and 2-methylcyclohexenone substrate **2a**, the reaction was set up with 50 mM MOPS-NaOH buffer pH 7.0, 2 µM *Ts*OYE, 1 mM $NADP^+$, 2 mg lyophilised GDH-101 (20.5 U/mg), 27.7 mM glucose, 10 mM 2-methylcyclohexenone, 1 mL volume, at 30 °C and 900 rpm in an Eppendorf Thermomixer C for 24 h, obtaining >99.9% conversion and 85.5% *ee* (see Figure S13).

Table S2. Conversions for the reduction of cyclohexenone **1a** to cyclohexanone **3a** catalysed by free *Ts*OYE in organic solvents.[a]

**Reaction media (91.7% v/v)**	**Water (% v/v)**	**Conv. 1a to 1b (%)**
Toluene	8.3	85.6 ± 0.6
EtOAc	8.3	86.8 ± 1.8
MTBE	8.3	>99.9
Buffer saturated MTBE	8.3	>99.9
Heptane	8.3	>99.9

[a] Conditions: 10 mmol cyclohexenone, 10 U/mL *Bs*GDH, 20 mmol glucose, 0.2 mmol NADPH, 1.4 µM *Ts*OYE, shaken at 30 °C and 900 rpm (Eppendorf ThermoMixer C) for 24 h. Buffer: 50 mM MOPS-NaOH pH 7.0.

Immobilised enzyme

For reactions with 15 mg of immobilised *Ts*OYE on Celite 545 (**Table S3** entries 1-3), the reactions were set up with the corresponding organic solvent, 0.2 mmol NADPH, 10 U *Bs*GDH, anhydrous glucose, 10 mmol cyclohexenone **1a**, 1 mL in volume, at 30 °C with the specified conditions shown in **Table S3**.

For reactions with immobilised *Ts*OYE on Celite 545, R-632, R-633, and R-648, the reactions were set up with the corresponding organic solvent, hydrated salt pairs ($Na_2HPO_4{\cdot}12H_2O$/ $Na_2HPO_3{\cdot}5H_2O$, 1:1 w/w), the specified amount of $NADP^+$, 2 mg GDH-101 (20.5 U/mg), anhydrous glucose, substrate, 1 mL in volume, at 30 °C with the specified conditions shown in **Table S3**.

As a control (**Table S3** entry 4), buffer saturated MTBE (50 mM MOPS-NaOH pH 7.0) was used in the reaction conditions described with: 10 mmol cyclohexenone, 1 mmol $NADP^+$, 27.7 mmol glucose, 2 mg GDH-101 and 15 mg immobilised *Ts*OYE on Celite R-633.

Table S3. Reaction conditions for cyclohexenone **1a** or 2-methyl-*N*-phenylmaleimide **3a** reduction catalysed by immobilised *Ts*OYE on Celite in organic solvent [a]

Entry	Organic solvent	Cofactor	[Cofactor] (mM)	[Glucose] (mmol)	Salt pairs (mg)	Buffer (% v/v)	Celite carrier	Celite *Ts*OYE amount (mg)	Time (h)	Conv. (%)	*ee* (%)
1[b]	-	NADPH	0.2	20	-	100	545	15	24	>99.9	
2[b]	EtOAc	NADPH	0.2	20	-	7.3	545	15	24	>99.9	-
3[b]	MTBE	NADPH	0.2	20	-	7.3	545	15	24	>99.9	-
4[b]	MTBE	$NADP^+$	1	27.7	25	1	545	50	8	91.0 ± 1.4	-
5[b]	MTBE	$NADP^+$	1	27.7	25	1	R-633	40	6	80.9 ± 0.1	-
6[b]	MTBE	$NADP^+$	1	27.7	25	1	R-633	40	24	98.7 ± 0.4	-
7[b]	Limonene	$NADP^+$	1	27.7	25	1	R-633	40	24	94.5 ± 4.9	-
8[b]	MTBE	$NADP^+$	1	27.7	25	1	R-632	40	24	98.8 ± 0.3	-
9[c]	MTBE	$NADP^+$	1	27.7	25	4	R-633	40	24	71.7 ± 6.6	>99.9
10[c,d]	MTBE	$NADP^+$	1	27.7	25	4	R-633	40	24	>99.9	>99.9
11[c]	MTBE	$NADP^+$	1	27.7	-	7	R-633	50	6	77.5 ± 3.5	>99.9

[a] Conditions: *Ts*OYE immobilised on Celite, 2 mg GDH-101 (20.5 U/mg), anhydrous glucose, cofactor, hydrated salt $Na_2HPO_4{\cdot}12H_2O$/ $Na_2HPO_3{\cdot}5H_2O$ (1:1 w/w), buffer (50 mM MOPS-NaOH pH 7.0), MTBE, substrate added with 1% v/v DMSO, 1 mL volume, 24 h at 30 °C, 900 rpm in an Eppendorf Thermomixer C; [b] 10 mM cyclohexenone **1a**; [c] 10 mM 2-methyl-*N*-phenylmaleimide **3a**; [d] Shaking with New Brunswick Scientific Excella E24 Incubator Shaker Series, 180 rpm.

Control experiments

Control experiments (**Table S4**) were performed with cyclohexenone as substrate in absence of enzymes. Tubes were shaken for 6, 8 or 24 h at 30 °C and 900 rpm in an Eppendorf ThermoMixer C. Aliquots (100 µL) were taken from the organic supernatant, diluted in 50 µL of EtOAc supplemented with 5 mM of tridecane as internal standard, dried with anhydrous $MgSO_4$, centrifuged, and transferred into GC vials for analysis.

Table S4. Reaction conditions for control reactions without *Ts*OYE in MTBE. [a]

[GDH-101] (U/mg)	Celite carrier	Celite *Ts*OYE amount (mg)	Conv. (%)
-	-	-	<0.1
20.5	-	-	<0.1
20.5	545	20	<0.1
20.5	R-632	20	<0.1
20.5	R-633	20	<0.1
20.5	R-648	20	<0.1

[a] Conditions: 10 mmol cyclohexenone, 27.7 mmol glucose, 1 mmol $NADP^+$, shaken at 30 °C and 900 rpm (Eppendorf ThermoMixer C) for 6 h.

To determine whether GDH-101 could reduce either the substrate cyclohexenone or product cyclohexanone under the same reaction conditions, control experiments were performed:
1) 10 mmol of cyclohexenone, 1 mmol $NADP^+$, 27.7 mmol glucose and 2 mg GDH-101 in MTBE. No conversion was observed.
2) 10 mmol of cyclohexanone, 1 mmol $NADP^+$, 27.7 mmol glucose and 2 mg GDH-101 in MTBE. 1% of cyclohexanol was observed.

Scale-up

Scale up reactions with 50 mmol 2-methyl-*N*-phenylmaleimide were set up with MTBE solvent, 9.3 mg substrate, 50 mg immobilised *Ts*OYE on Celite R-633 and R-648, 2 mmol of $NADP^+$, 2 mg GDH-101 and 60 mmol of glucose, 1 mL in volume. The vials were shaken for 24 h at 30 °C and 180 rpm on an incubator shaker (New Brunswick Scientific Excella E24 Incubator Shaker Series). Aliquots (100 µL) were taken from the organic supernatant, diluted in 50 µL of EtOAc supplemented with 5 mM of tridecane as internal standard, dried with anhydrous $MgSO_4$, centrifuged, and transferred into GC vials for analysis.
For **Table 2** entries 19 and 20, the pure product was separated from the organic solvent by evaporation of the solvent and the solid product was obtained in 91% isolated yield in both cases, and analysed by NMR in DMSO-d_6 (**Figures S17** and **S18** for the R-633-*Ts*OYE reaction). Spectra are in agreement with literature.[6]

Immobilised enzyme recovery

Enzyme reusability was determined through several cycles of cyclohexanone synthesis. The reaction mixture contained: 10 mmol cyclohexenone, 1 mmol $NADP^+$, 27.7 mmol solid anhydrous glucose, 2 mg GDH-101, 25 mg of salt pairs ($Na_2HPO_4 \cdot 12H_2O/Na_2HPO_3 \cdot 5H_2O$, 1:1 w/w), 50 mg of immobilised *Ts*OYE on Celite R-633 or Celite 545, and 0.98 mL MTBE. The reaction mixture was incubated and shaken at 900 rpm and 30 °C for 24 h. At the end of the reaction, the liquid mixture was separated from the immobilised enzyme by centrifugation and a new reaction mixture was prepared for the next operational cycle.

Product analyses

Gas chromatography analyses were performed on a GC-2010-Plus apparatus (Shimadzu Europe, Germany) equipped with a flame ionization detector (FID) and a CP-Sil 8 CB (50 m × 0.53 mm × 1.0 µm). The substrate and product were analysed under the following conditions: N_2 carrier gas at 20 mL/min;

injector temperature at 340 °C; oven program: 80 °C for 3 min; 20 °C/min to 340 °C for 1 min; detector temperature at 360 °C.
Peak retention times (min) were as follows: cyclohexanone 3.07; cyclohexenone 3.56; tridecane 7.30; 2-methyl-*N*-phenylmaleimide 9.46; 2-methyl-*N*-phenylsuccimide 9.82.

For the analysis of (*R*)-2-methylcyclohexanone **2b**, the column CP-Chirasil-DEX CB (25 m × 0.32 mm × 0.25 µm) was used. The substrate and the product were analysed under the following conditions: N_2 carrier gas at 30 mL/min; injector temperature at 250 °C; oven program: 70 °C for 2 min; 5 °C/min to 80 °C for 3 min; 5 °C/min to 90 °C for 3 min; 5 °C/min to 100 °C for 2 min; 10 °C/min to 220 °C for 1 min; detector temperature at 275 °C.
Peak retention times (min) were as follows: 2-methylcyclohexenone 12.5; (*R*)-2-methylcyclohexanone 11.3; (*S*)-2-methylcyclohexanone 11.1.
Please note that the commercially available substrate 2-methylcyclohexenone (CAS 1121-18-2) obtained from Sigma-Aldrich (771368) is of ≥90% purity only and contains ~5% of the isomer 6-methylcyclohexenone (GC retention time 12.6 min), which is accepted as a substrate by *Ts*OYE and thus eventually produces a 5% racemic mixture of 2-methylcyclohexanone, leading to 2.5% of the undesired (*S*)-2-methylcyclohexanone.

For the analysis of (*R*)-2-methyl-*N*-phenylsuccinimide **3b**, the column Hydrodex β-TBDAc (50 m × 0.25 mm × 0.25 µm) was used. The substrate and the product were analysed under the following conditions: N_2 carrier gas at 30 mL/min; injector temperature at 250 °C; oven program: 70 °C for 3 min; 5 °C/min to 90 °C for 3 min; 10 °C/min to 180 °C for 3 min; 5 °C/min to 190 °C for 8 min; 5 °C/min to 200 °C for 8 min; 10 °C/min to 220 °C for 2 min; detector temperature at 260 °C.
Peak retention times (min) were as follows: tridecane 17.6; 2-methyl-*N*-phenylmaleimide 33.1; (*R*)-2-methyl-*N*-phenylsuccimide 37.6. The (*R*)-configuration was assumed based on literature with the same enzyme and product.[4, 7]

GC chromatograms

GC chromatograms obtained on the column CP-Sil 8 CB (50 m × 0.53 mm × 1.0 µm):

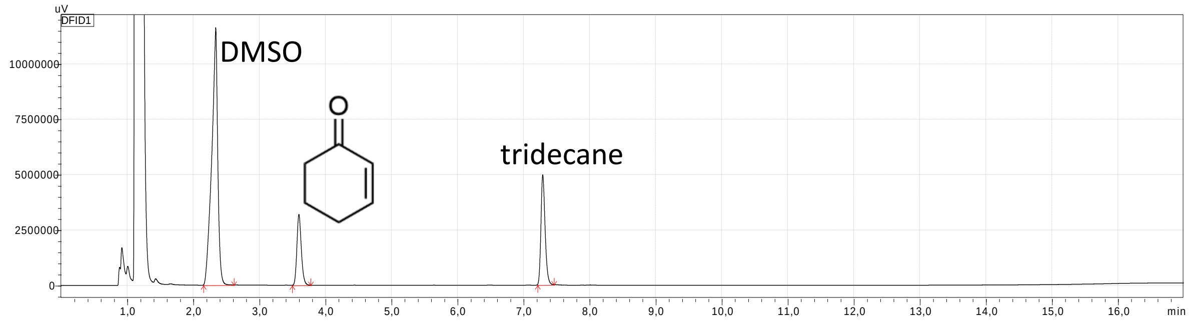

Figure S1. GC chromatogram of cyclohexenone **1a** standard with DMSO and tridecane.

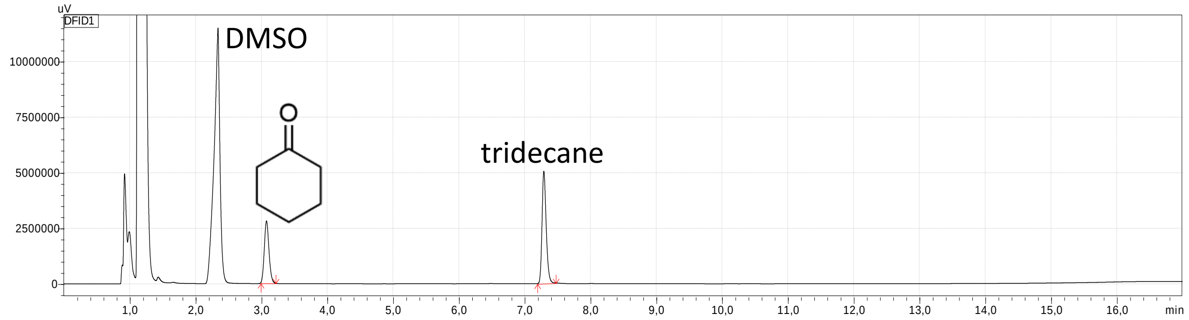

Figure S2. GC chromatogram of cyclohexanone **1b** standard with DMSO and tridecane.

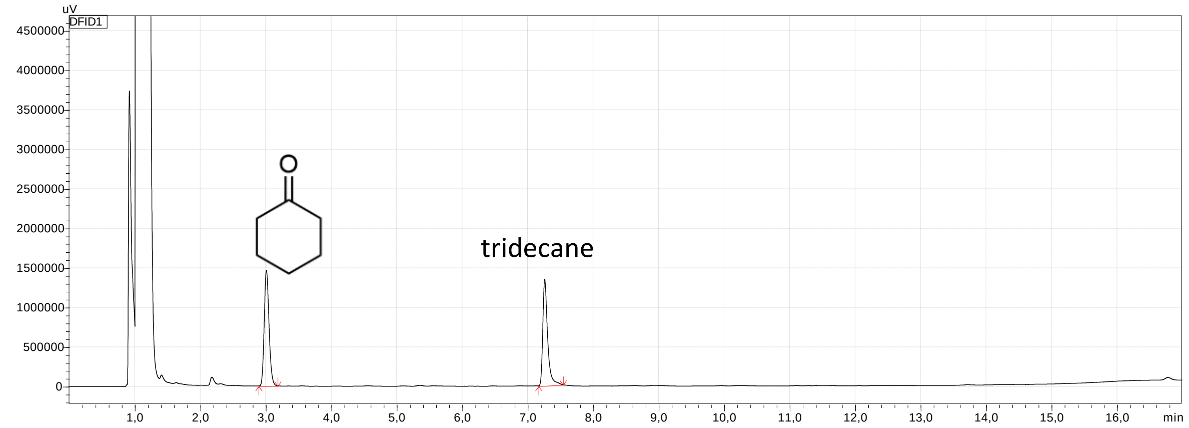

Figure S3. GC chromatogram of cyclohexenone **1a** reduction catalysed by free *Ts*OYE in buffer.

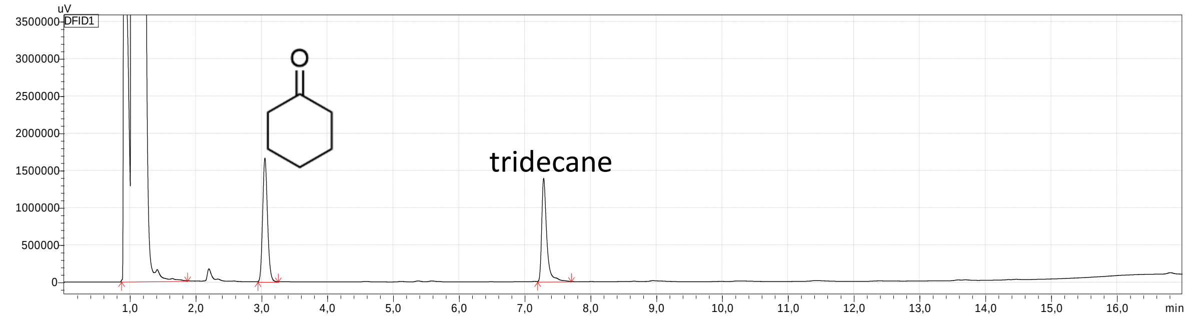

Figure S4. GC chromatogram of cyclohexenone **1a** reduction catalysed by *Ts*OYE on Celite 545.

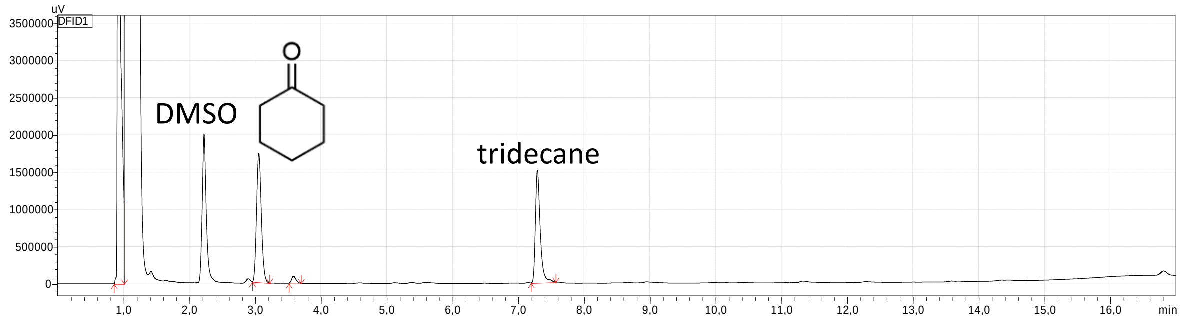

Figure S5. GC chromatogram of cyclohexenone **1a** reduction catalysed by *Ts*OYE on Celite R-632 (Table 2 entry 3).

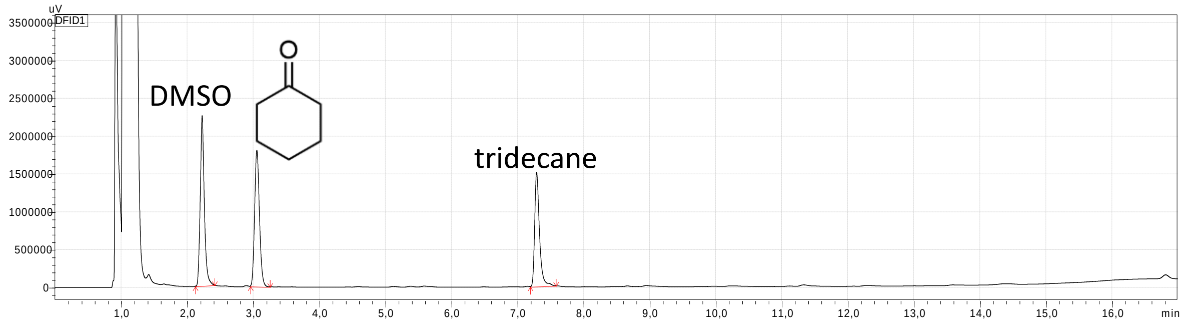

Figure S6. GC chromatogram of cyclohexenone **1a** reduction catalysed by *Ts*OYE on Celite R-633 (Table 2 entry 4).

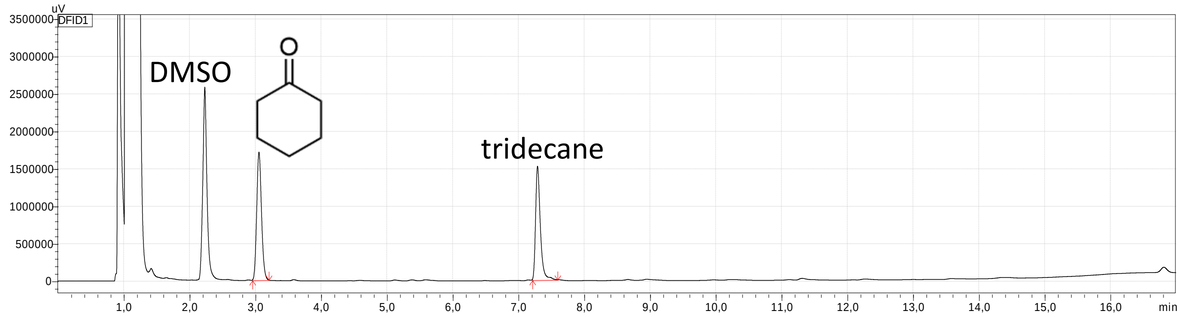

Figure S7. GC chromatogram of cyclohexenone **1a** reduction catalysed by *Ts*OYE on Celite R-648 (Table 2 entry 5).

Chiral GC chromatograms obtained on the column CP-Chirasil-DEX CB (25 m × 0.32 mm × 0.25 μm):

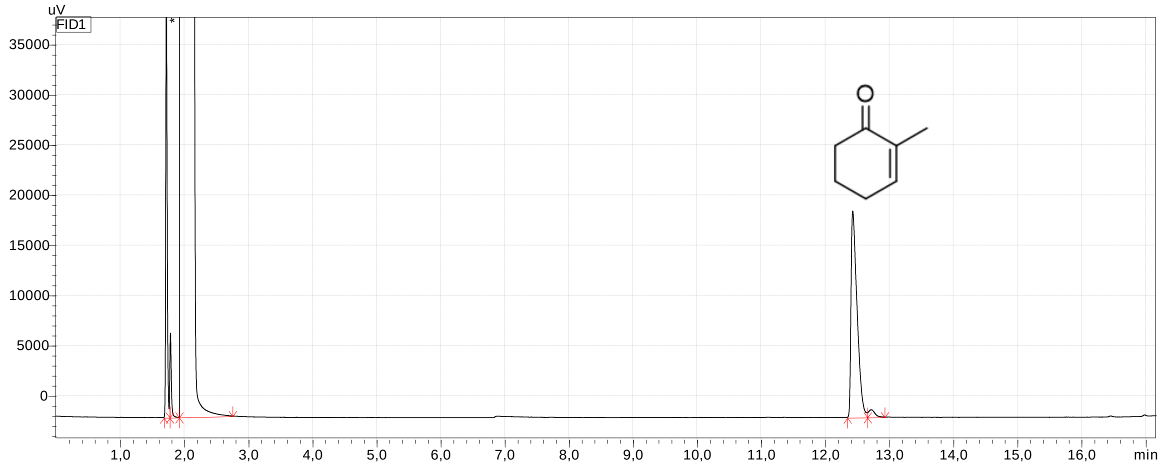

Figure S8. GC chromatogram of 2-methylcyclohexenone **2a** standard.

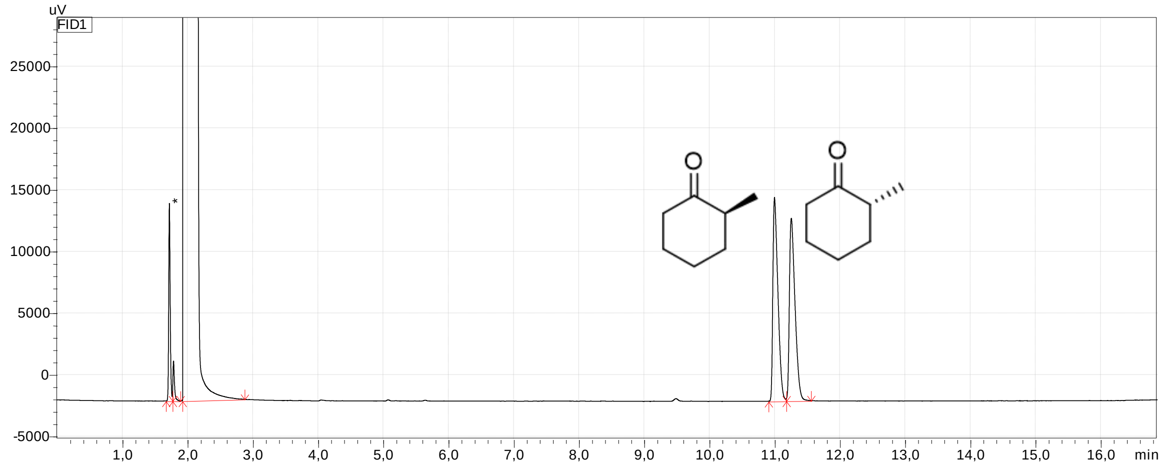

Figure S9. GC chromatogram of racemic 2-methylcyclohexanone **2b** standard.

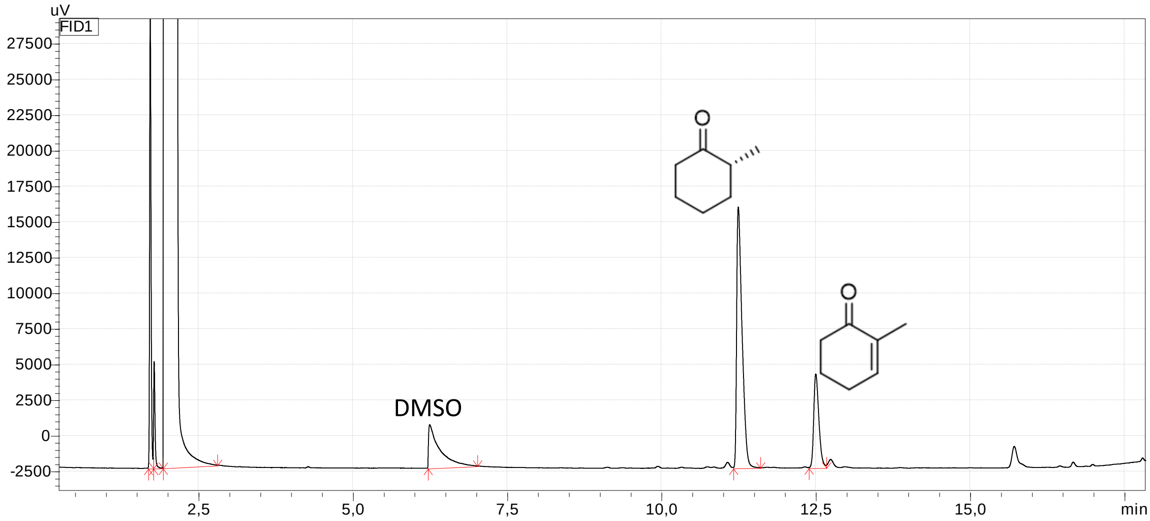

Figure S10. GC chromatogram of 2-methylcyclohexenone **2a** reduction catalysed by *Ts*OYE on Celite 545 (Table 2, entry 11).

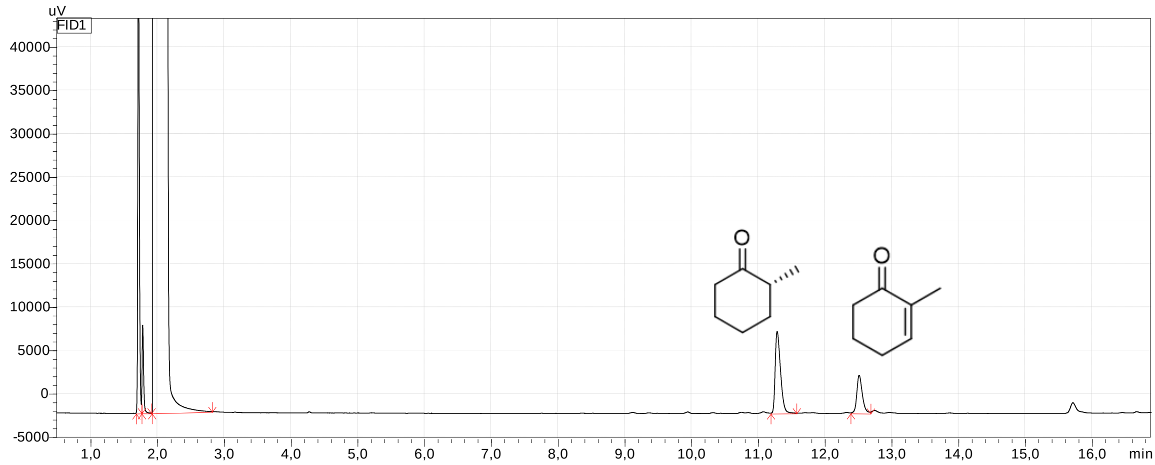

Figure S11. GC chromatogram of 2-methylcyclohexenone **2a** reduction catalysed by *Ts*OYE on Celite 545, 2% v/v buffer content (Table 2, entry 12).

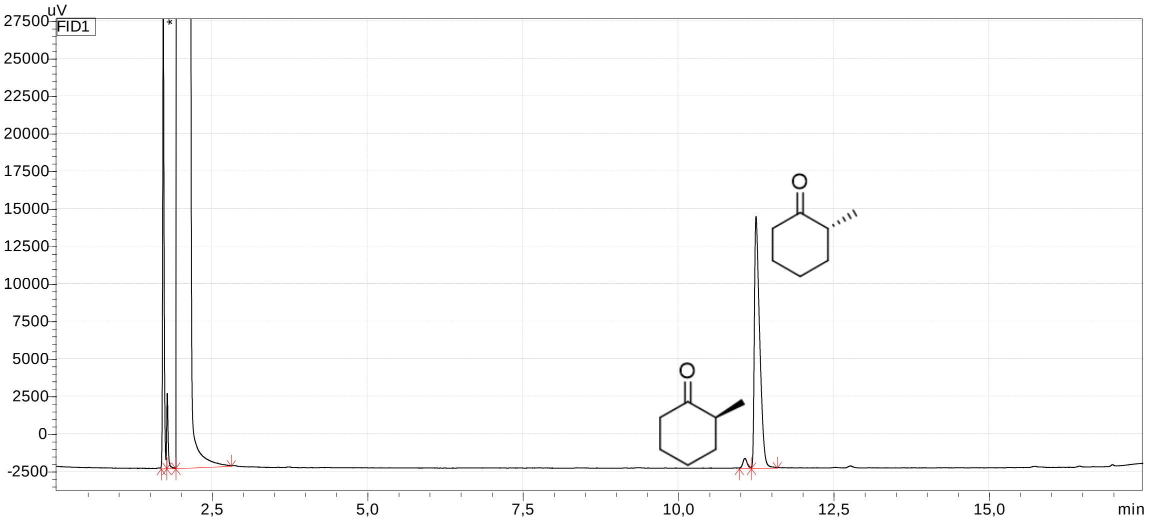

Figure S12. GC chromatogram of 2-methylcyclohexenone reduction catalysed by *Ts*OYE on Celite 545 (Table 2, entry 13).

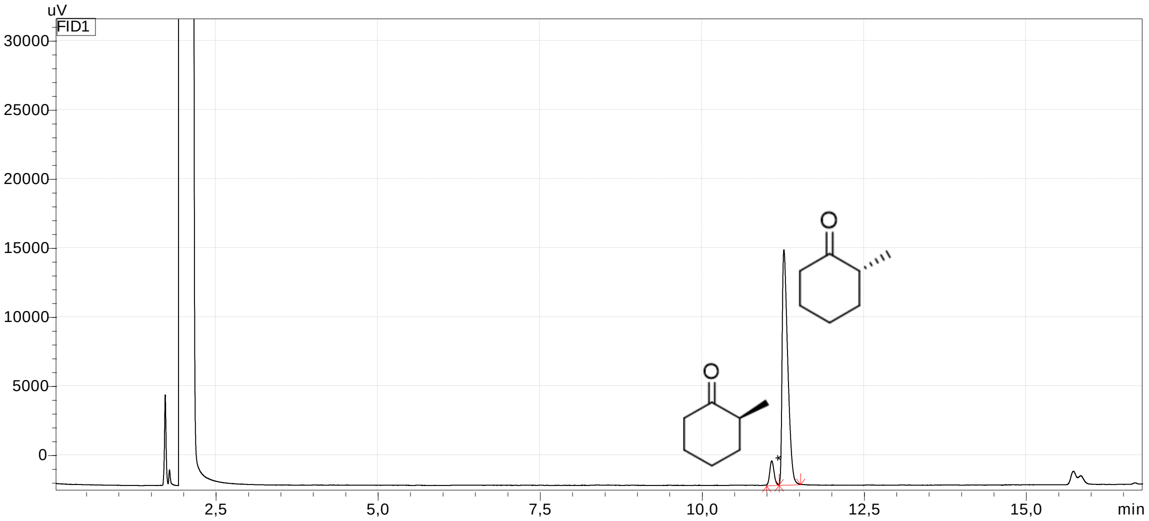

Figure S13. GC chromatogram of 2-methylcyclohexenone reduction catalysed by free *Ts*OYE in 50 mM MOPS-NaOH pH 7.0 buffer.

GC chromatograms obtained on the column Hydrodex β-TBDAc (50 m × 0.25 mm × 0.25 µm):

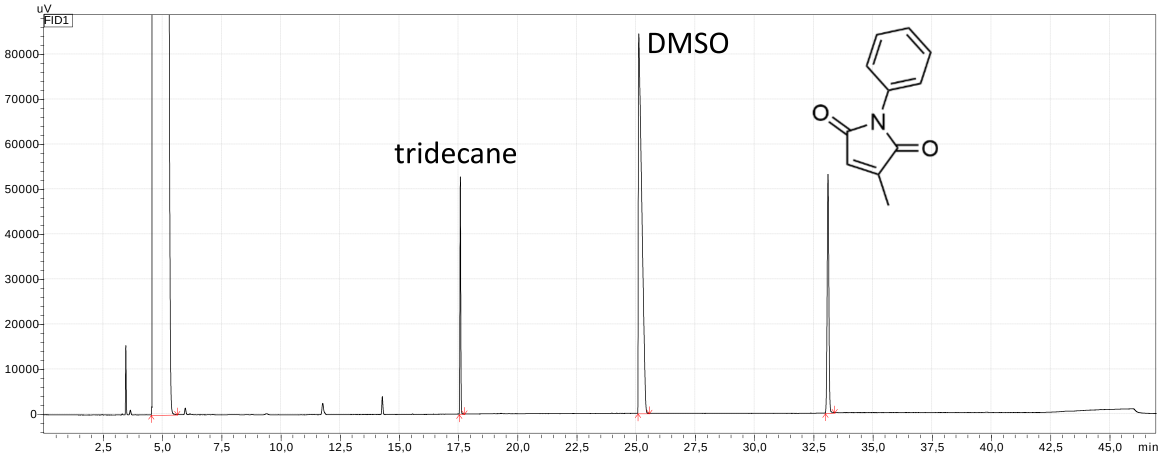

Figure S14. GC chromatogram of 2-methyl-*N*-phenylmaleimide **3a** standard with DMSO and tridecane. Note impurity peaks at 11.9 and 14.4 min are from the GC column itself.

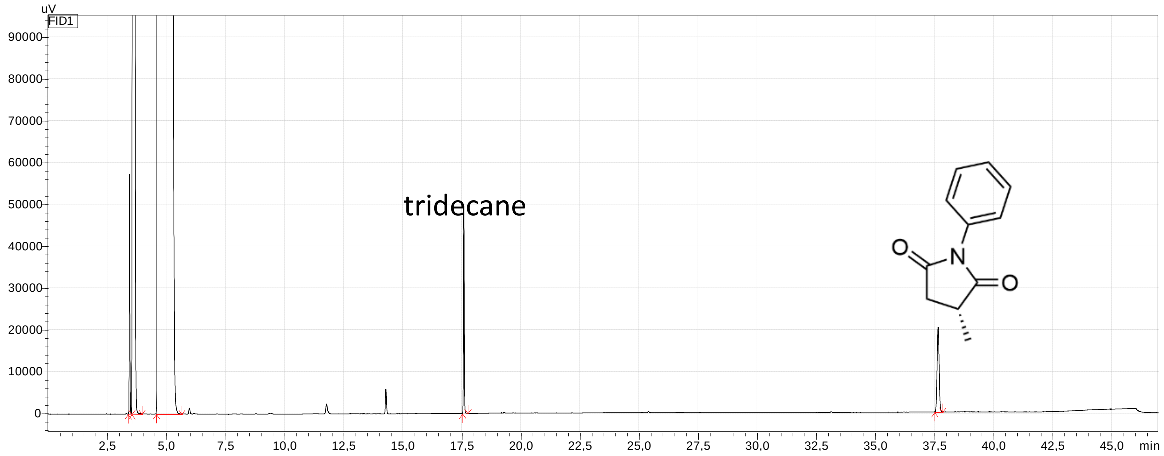

Figure S15. GC chromatogram of 2-methyl-*N*-phenylmaleimide **3a** reduction to 2-methyl-*N*-phenylsuccinimide **3b** catalysed by *Ts*OYE on Celite R-633 (Table 2 entry 19). Note impurity peaks at 11.9 and 14.4 min are from the GC column (see **Figure S14**).

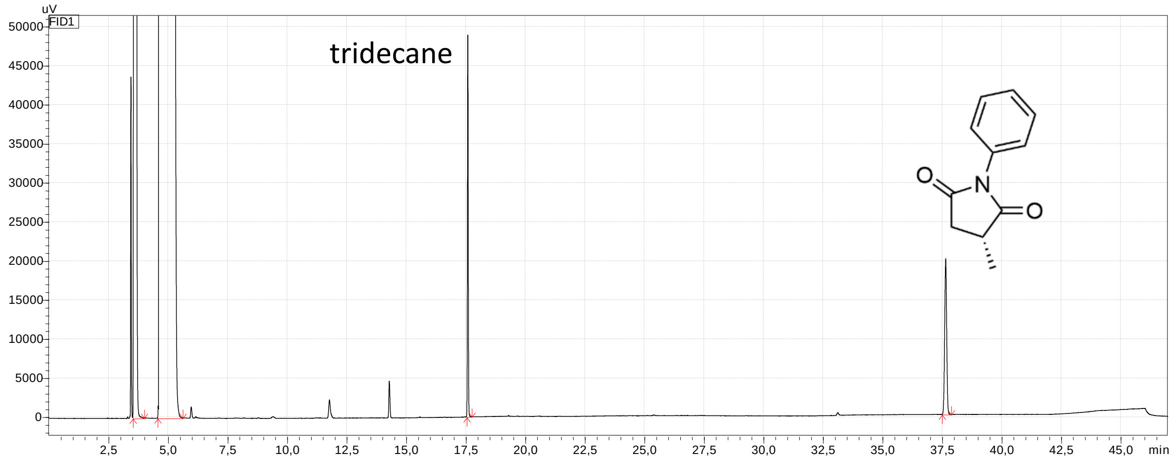

Figure S16. GC chromatogram of 2-methyl-*N*-phenylmaleimide **3a** reduction to 2-methyl-*N*-phenylsuccinimide **3b** catalysed by *Ts*OYE on Celite R-648 (Table 2 entry 20). Note impurity peaks at 11.9 and 14.4 min are from the GC column (see **Figure S14**).

^{1}H-NMR and ^{13}C-NMR spectra of isolated (*R*)-2-methyl-*N*-phenylsuccinimide product:

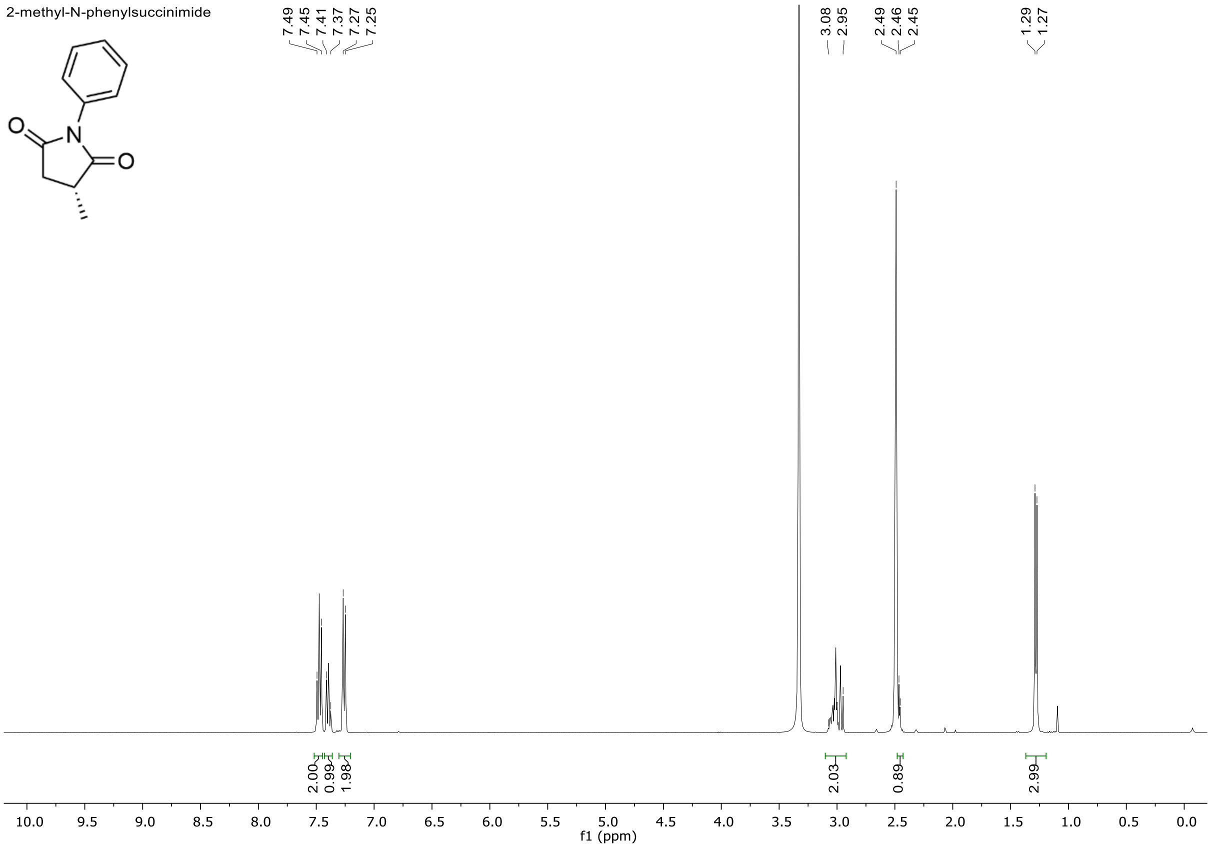

Figure S17. ^{1}H-NMR spectrum (400 MHz, DMSO-d_6), δ (ppm): 7.49-7.45 (m, 2H), 7.41-7.37 (m, 1H), 7.27-7.25 (m, 2H), 3.08-2.95 (m, 2H), 2.46-2.45 (m, 1H), 1.29-1.27 (d, 3H). Spectra are in accordance with literature.[6]

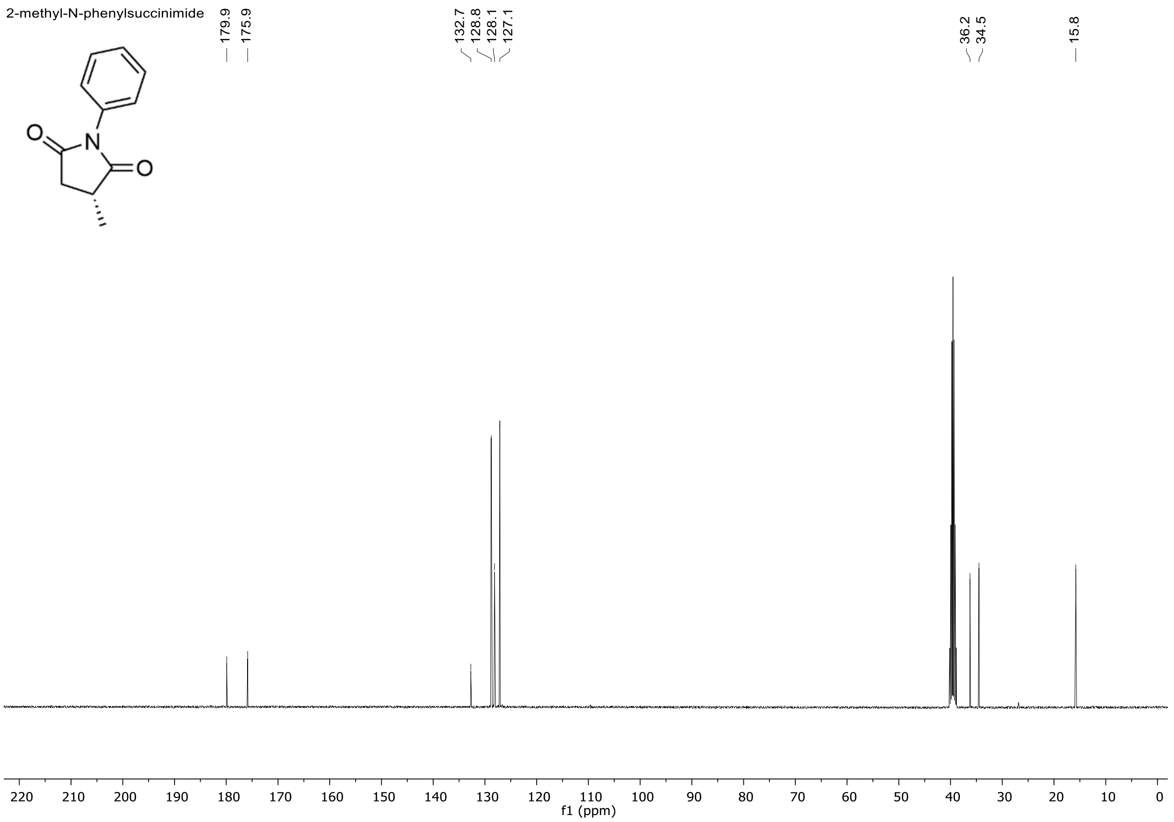

Figure S18. ^{13}C-NMR spectrum (100 MHz, DMSO-d_6), δ (ppm): 179.9, 175.9, 132.7, 128.8, 128.1, 127.1, 36.2, 34.5, 15.8.

References

1 M. M. Bradford, *Anal. Biochem.*, 1976, **72**, 248-254.
2 A. Aliverti, B. Curti and M. A. Vanoni, in *Flavoprotein Protocols*, eds. S. K. Chapman and G. A. Reid, Humana Press, Totowa, NJ, 1999, pp. 9-23.
3 A. Riedel, M. Mehnert, C. E. Paul, A. H. Westphal, W. J. H. van Berkel and D. Tischler, *Front. Microbiol.*, 2015, **6**, 1073.
4 A. Scholtissek, S. R. Ullrich, M. Muhling, M. Schlomann, C. E. Paul and D. Tischler, *Appl. Microbiol. Biotechnol.*, 2017, **101**, 609-619.
5 J. Coloma, L. Teeuwisse, M. Afendi, P.-L. Hagedoorn and U. Hanefeld, *Catalysts*, 2022, **12**, 161.
6 T. Hirata, A. Takarada, M. E. F. Hegazy, Y. Sato, A. Matsushima, Y. Kondo, A. Matsuki and H. Hamada, *J. Mol. Catal. B: Enzym.*, 2005, **32**, 131-134.
7 C. E. Paul, S. Gargiulo, D. J. Opperman, I. Lavandera, V. Gotor-Fernández, V. Gotor, A. Taglieber, I. W. C. E. Arends and F. Hollmann, *Org. Lett.*, 2013, **15**, 180-183.
